# Lidocaine enhances the effects of chemotherapeutic drugs against bladder cancer

**DOI:** 10.1038/s41598-017-19026-x

**Published:** 2018-01-12

**Authors:** Xihua Yang, Lili Zhao, Meiping Li, Lei Yan, Shengwan Zhang, Zhenguo Mi, Liansheng Ren, Jun Xu

**Affiliations:** 1 Affiliated Cancer Hospital, Shanxi Medical University, Taiyuan, Shanxi China; 2 College of Life Science, Shanxi University, Taiyuan, Shanxi China

## Abstract

This study aimed to investigate whether lidocaine, alone or in combination with other chemotherapeutic agents, inhibits the growth of human bladder cancer cells *in vitro* and orthotopically transplanted bladder tumors *in vivo*. The effects of lidocaine (1.25, 2.5 or 5 mg/mL), mitomycin C (MMC, 0.66 mg/mL), pirarubicin (0.75 mg/mL) and Su Fu’ning lotion (SFN, 0.0625 mg/mL) on the proliferation of human bladder cancer (BIU-87) cells were studied using the MTT assay. A Balb/c nude mouse model of bladder cancer was developed by orthotopic transplantation of BIU-87 cells, and the effects of intravesical instillation of lidocaine and MMC on bladder wet weight (a measure of tumor size) and survival (over 60 days) were studied. Lidocaine inhibited proliferation of BIU-87 cells in a concentration-dependent manner and (when given in combination) enhanced the actions of each of the other antiproliferative agents. In tumor-bearing mice, MMC alone had no effect on mean survival or bladder wet weight. However, the combination of 0.66 mg/mL MMC and 5 mg/mL lidocaine prolonged survival (from 34.62 ± 6.49 to 49.30 ± 6.72 days; n = 8, *P* < 0.05) and reduced bladder wet weight (from 68.94 ± 53.61 to 20.26 ± 6.07; n = 8, *P* < 0.05). Intravesical instillation of lidocaine combined with other chemotherapeutic agents potentially could be an effective therapy for bladder cancer.

## Introduction

Bladder cancer is one of the most common malignant tumors in humans and is associated with a high mortality. In 2012, there were approximately 430,000 new cases of bladder cancer worldwide, and the disease accounted for over 165,000 deaths^1^. The majority of cases of bladder cancer occur in men, and smoking is recognized as an important risk factor^2^. Approximately two-thirds of patients with bladder cancer present with superficial tumors, while one-third present with a more invasive form of the disease that is associated with a high risk of distant metastasis^3^. The management of bladder cancer includes intravesical therapy (for tumors that have not invaded the muscle), transurethral resection of bladder tumor (TURBT), radical cystoprostatectomy, radiotherapy and chemotherapy^3^. Despite recent advances in the detection and treatment of bladder cancer^4^, the high recurrence and mortality rates associated with bladder cancer^1^ highlight the need for improvements in the diagnosis and treatment of this disease.

In patients who undergo surgery for bladder cancer, postoperative instillation of drugs into the bladder plays an important role in the removal of residual cancer cells and decreases the recurrence rate. At present, several drugs are commonly used for intravesical instillation, such as pirarubicin, epirubicin, mitomycin C (MMC) and hydroxycamptothecine. A recent meta-analysis has demonstrated that intravesical administration of pirarubicin, epirubicin and MMC after TURBT can reduce tumor recurrence^5^. However, a notable drawback of these drugs is their serious toxic and adverse actions (including chemical cystitis and cystospasm)^6,7^. Another drawback is the limited treatment effect, with recurrence rates reported to be as high as 30–50% within 2 years of the administration of these agents^2,3^. Therefore, the development of new anticancer drugs for instillation into the bladder after surgery for bladder cancer would substantially improve the treatment of this disease.

Lidocaine is mainly used as a local anesthetic, but it also has additional non-anesthetic effects. Numerous studies in recent years have found that lidocaine can inhibit the inflammatory reaction^4,8^, protect against acute lung injury^5–7^, exert a neuroprotective effect and alleviate postoperative cognitive dysfunction (POCD)^9–11^. There is also evidence that lidocaine may have anticancer actions in a wide range of cancer cells^12–16^, and it may have a sensitizing effect on anticancer drugs^17,18^. Procaine, another local anesthetic agent, has been found to attenuate the growth of MCF-7 breast cancer cells^19^. To date, no studies have investigated whether lidocaine can affect the viability, invasiveness and/or migration of bladder cancer cells. We hypothesized that lidocaine might inhibit the proliferation of bladder cancer cells, and its potential anticancer effects might be increased in combination with chemotherapy.

Therefore, the aim of the present study was to investigate whether lidocaine, alone or in combination with other chemotherapeutic agents, would inhibit the proliferation of a human bladder cancer cell line and, when administered by intravesical instillation, attenuate the growth of orthotopically transplanted bladder tumors in mice.

## Results

### Inhibition of BIU-87 cell proliferation by lidocaine, MMC or their combination

Lidocaine alone significantly inhibited the proliferation of BIU-87 cells (assessed using the MTT assay) as compared with the control group, with cell inhibition rates of 23.64% for 1.25 mg/mL lidocaine, 45.88% for 2.5 mg/mL lidocaine and 75.49% for 5 mg/mL lidocaine (all *P*0.05 versus the control group; Table 1). The cell inhibition rate for MMC alone (0.66 mg/mL) was 88.94% (*P* < 0.05 versus the control group). Compared with MMC alone, the cell inhibition rates were significantly higher when MMC (0.66 mg/mL) was combined with lidocaine: the cell inhibition rates were 92.95% for MMC plus 1.25 mg/mL lidocaine, 95.12% for MMC plus 2.5 mg/mL lidocaine and 97.18% for MMC plus 5 mg/mL lidocaine (all *P* < 0.05 versus MMC alone; Table 1). This indicates that the combination of lidocaine and MMC has a synergistic effect to inhibit the proliferation of BIU-87 cells.

**Table 1:** Inhibition of BIU-87 cell proliferation by lidocaine alone, MMC alone or the combination of both drugs.

Group	OD value	Cell inhibition rate (%)
Control	0.922 ± 0.014	—
1.25 mg/mL lidocaine	0.704 ± 0.022*	23.64
2.5 mg/mL lidocaine	0.499 ± 0.014*	45.88
5 mg/mL lidocaine	0.226 ± 0.014*	75.49
0.66 mg/mL MMC	0.102 ± 0.011*	88.94
0.66 mg/mL MMC + 1.25 mg/mL lidocaine	0.065 ± 0.006*^,▲^	92.95
0.66 mg/mL MMC + 2.5 mg/mL lidocaine	0.045 ± 0.008*^,▲^	95.12
0.66 mg/mL MMC + 5 mg/mL lidocaine	0.026 ± 0.012*^,▲^	97.18

Note: MMC, mitomycin C; OD, optical density. *P < 0.05 versus control; ^▲^*P* < 0.05 versus MMC alone.

### Inhibition of BIU-87 cell proliferation by lidocaine, pirarubicin or their combination

In this set of experiments, the cell inhibition rates for lidocaine alone were 22.31% for 1.25 mg/mL lidocaine, 46.18% for 2.5 mg/mL lidocaine and 73.55% for 5 mg/mL lidocaine (all *P* < 0.05 versus the control group; Table 2). The cell inhibition rate for pirarubicin alone (0.75 mg/mL) was 76.24% (*P* < 0.05 versus the control group). The addition of lidocaine to pirarubicin (0.75 mg/mL) caused a further reduction in BIU-87 cell proliferation: the cell inhibition rates were 81.82% for pirarubicin plus 1.25 mg/mL lidocaine, 86.16% for pirarubicin plus 2.5 mg/mL lidocaine and 96.90% for pirarubicin plus 5 mg/mL lidocaine (all *P* < 0.05 versus pirarubicin alone; Table 2). Thus, the combination of lidocaine and pirarubicin was more effective than pirarubicin alone at inhibiting BIU-87 cell proliferation.

**Table 2:** Inhibition of BIU-87 cell proliferation by lidocaine alone, pirarubicin alone or the combination of both drugs.

Group	OD value	Cell inhibition rate (%)
Control	0.968 ± 0.031	—
1.25 mg/mL lidocaine	0.752 ± 0.019*	22.31
2.5 mg/mL lidocaine	0.521 ± 0.024*	46.18
5 mg/mL lidocaine	0.256 ± 0.008*	73.55
0.75 mg/mL pirarubicin	0.230 ± 0.009*	76.24
0.75 mg/mL pirarubicin + 1.25 mg/mL lidocaine	0.176 ± 0.013*^,▲^	81.82
0.75 mg/mL pirarubicin + 2.5 mg/mL lidocaine	0.134 ± 0.005*^,▲^	86.16
0.75 mg/mL pirarubicin + 5 mg/mL lidocaine	0.030 ± 0.007*^,▲^	96.90

Note: OD, optical density. *P < 0.05 versus control; ^▲^*P* < 0.05 versus pirarubicin alone.

### Inhibition of BIU-87 cell proliferation by lidocaine, SFN or their combination

The cell inhibition rates for lidocaine alone were 26.81% for 1.25 mg/mL lidocaine, 41.80% for 2.5 mg/mL lidocaine and 77.64% for 5 mg/mL lidocaine; the corresponding value for 0.0625 mg/mL SFN was 57.69% (all *P* < 0.05 versus the control group; Table 3). Combining lidocaine with SFN resulted in a greater attenuation of BIU-87 cell proliferation than SFN alone: the cell inhibition rates were 85.13% for SFN plus 1.25 mg/mL lidocaine, 88.31% for SFN plus 2.5 mg/mL lidocaine and 93.01% for SFN plus 5 mg/mL lidocaine (all *P* < 0.05 versus SFN alone; Table 3). Therefore, lidocaine and SFN had an additive effect on the inhibition of BIU-87 cell proliferation.

**Table 3:** Inhibition of BIU-87 cell proliferation by lidocaine alone, SFN alone or the combination of both drugs.

Group	OD value	Cell inhibition rate (%)
Control	0.787 ± 0.024	—
1.25 mg/mL lidocaine	0.576 ± 0.014*	26.81
2.5 mg/mL lidocaine	0.458 ± 0.013*	41.80
5 mg/mL lidocaine	0.176 ± 0.015*	77.64
0.0625 mg/mL SFN	0.333 ± 0.015*	57.69
0.0625 mg/mL SFN + 1.25 mg/mL lidocaine	0.117 ± 0.014*^,▲^	85.13
0.0625 mg/mL SFN + 2.5 mg/mL lidocaine	0.092 ± 0.009*^,▲^	88.31
0.0625 mg/mL SFN + 5 mg/mL lidocaine	0.055 ± 0.009*^,▲^	93.01

Note: SFN, Su Fu’ning lotion; OD, optical density. *P < 0.05 versus control; ^▲^*P* < 0.05 versus SFN alone.

### The effect of bladder perfusion with MMC or/and lidocaine on orthotopically transplanted bladder cancer in mice

**Figure 1:**
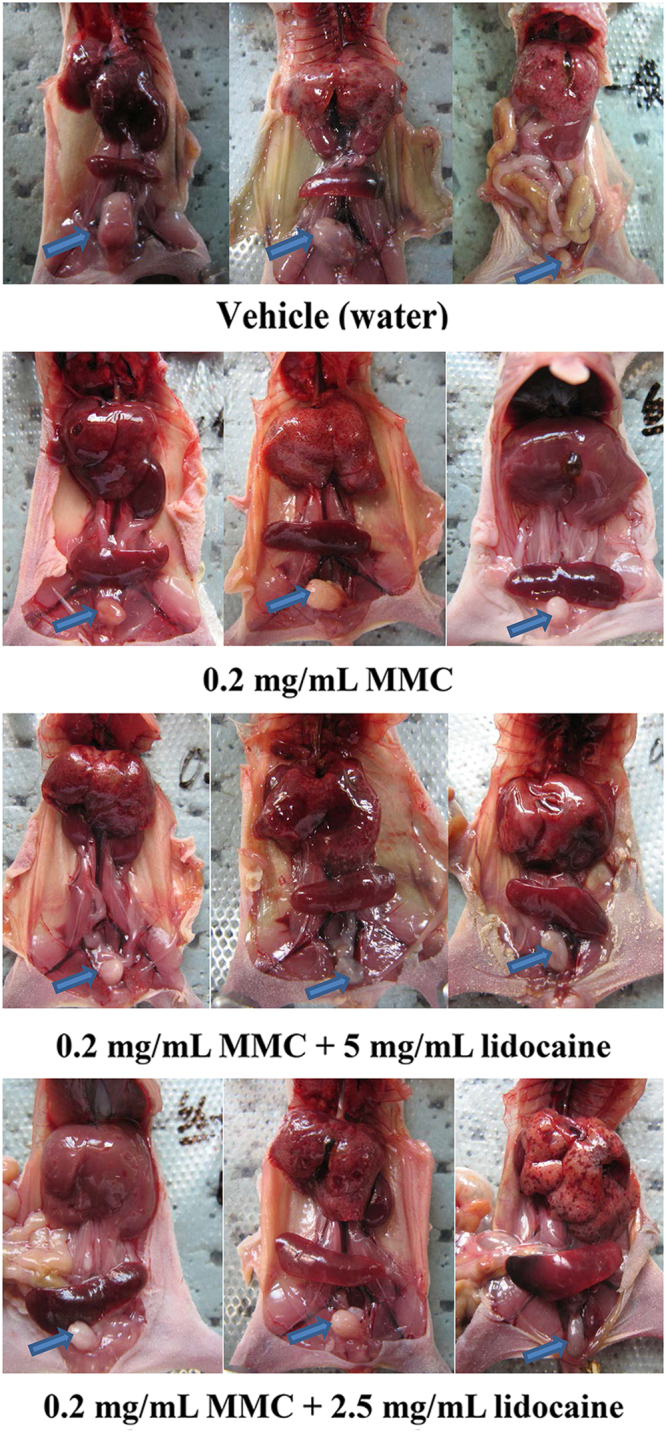
Representative photographs illustrating the gross anatomy of the mice in each experimental group. The arrows mark the location of the tumor. Representative of n = 8 per group.

At the end of the 60-day observation period, all mice had died in both the vehicle group and the MMC/high lidocaine group (i.e., 0.2 mg/mL MMC plus 5 mg/mL lidocaine), while only 1 mouse had survived in each of the MMC alone group (i.e., 0.2 mg/mL MMC) and MMC/low lidocaine group (i.e., 0.2 mg/mL MMC plus 2.5 mg/mL lidocaine). Figure 1 shows representative images to illustrate the gross anatomy of the BALB/c nude mice in each of the experimental groups. In the vehicle group, the bladders of all mice had developed tumors. In the MMC group, 1 mouse survived for only 1 day without the formation of any tumor visible by the naked eye, 1 mouse developed a thickened bladder wall, and a further 6 mice developed tumors in the bladder. In the MMC/high lidocaine group, 1 mouse did not develop any tumors visible by the naked eye, 3 mice developed a thickened bladder wall, and all the other mice developed tumors in the bladder. In the MMC/low lidocaine group, 2 mice did not develop any tumors visible by the naked eye, while the other mice developed tumors in the bladder; in 2 of these mice, the tumors were of mung bean size.

The mean survival time and mean bladder wet weight for each of the groups are shown in Table 4. Although survival time was numerically higher and bladder wet weight numerically lower in the MMC alone group than in the vehicle group, the differences were not statistically significant. However, survival time was significantly prolonged in the MMC/high lidocaine group compared with the vehicle group (*P* < 0.05; Table 4), while bladder wet weight was significantly lower in both the MMC/high lidocaine group and MMC/low lidocaine group than in the vehicle group (*P* < 0.05; Table 4). We found no evidence of lidocaine toxicity in any of the mice studied.

**Table 4:** Effect of MMC alone or in combination with lidocaine on the survival time and bladder wet weight of BALB/C nude mice with orthotopically transplanted bladder cancer.

Group	Survived 60 days (n/N)	Mean survival time (days)	Mean bladder wet weight (mg)	Tumor inhibition rate (%)	Life extension rate (%)
Vehicle	0/8	34.62 ± 6.49	68.94 ± 53.61	—	—
MMC	1/8	40.86 ± 9.15	36.15 ± 19.83	47.56	18.78
MMC/high lidocaine	0/8	49.30 ± 6.72*	20.26 ± 6.07*	70.61	42.40
MMC/low lidocaine	1/8	41.25 ± 12.92	19.80 ± 6.17*	71.28	19.15

Note: MMC, mitomycin C. MMC group: 0.2 mg/mL MMC alone; MMC/low lidocaine group: 0.2 mg/mL MMC plus 2.5 mg/mL lidocaine; MMC/high lidocaine group: 0.2 mg/mL MMC plus 5 mg/mL lidocaine. *P < 0.05 versus vehicle group.

## Discussion

The main finding of the present study was that lidocaine (1.25 to 5 mg/mL) inhibited the proliferation of BIU-87 bladder cancer cells in a concentration-dependent manner, and when given in combination enhanced the antiproliferative effects of MMC, pirarubicin and SFN. Furthermore, in mice with orthotopically transplanted bladder cancer, the combination of MMC and lidocaine (at 5 mg/mL) prolonged mean survival and reduced mean bladder wet weight. To the best of our knowledge, this is the first study to provide evidence that intravesical instillation of lidocaine in combination with other chemotherapeutic agents (such as MMC) could potentially be a more effective therapy for bladder cancer than monotherapy with one chemotherapeutic agent.

The clinical efficacy of pirarubicin and MMC is well established; for example, a recent meta-analysis concluded that intravesical administration of pirarubicin and MMC after TURBT was associated with a reduced tumor recurrence (hazard ratios of 0.31 and 0.40, respectively) and prolonged recurrence-free survival (hazard ratios of 0.31 and 0.44, respectively)^5^. Our finding that pirarubicin and MMC inhibited the proliferation of cultured BIU-87 cells is consistent with clinical observations and in agreement with other *in vitro* studies in bladder cancer cell lines^20–22^.

SFN is an extract from Sappan wood (*Caesalpinia sappan* L.) that is used as a traditional Chinese medicine. SFN can inhibit the proliferation of three different bladder cancer cell lines (BIU-87, T24 and BTT cells) as well as reduce bladder wet weight and prolong survival in mice with bladder cancer xenografts (using T24 or BTT cells)^20^. Although the mechanism by which SFN exerts an anticancer effect remains to be elucidated, our *in vitro* observations agree well with this previous study.

An important and novel finding of the present study was that lidocaine was able to attenuate the proliferation of BIU-87 cells and augment the antiproliferative actions of MMC, pirarubicin and SFN. Furthermore, the combination of lidocaine and MMC was able to prolong the survival of mice with orthotopic bladder cancer and reduce bladder wet weight, an indicator of tumor size. To our knowledge, no such effects of lidocaine on bladder cancer have been reported previously. Our observations are in good agreement with numerous studies demonstrating inhibitory effects of lidocaine on other types of cancer cells, including HT1080 (fibrosarcoma), HOS (osteosarcoma), RPMI-7951 (melanoma), LM8 (osteosarcoma), MDA-MB-231 (human breast cancer), PC-3 (prostatic cancer), ES-2 (ovarian cancer), NCI-H838 (lung cancer), CAL27 (tongue cancer), 8505 C (thyroid cancer) and K1 (thyroid cancer) cells^12–16^.

The present study was not designed to elucidate the mechanism by which lidocaine enhanced the effects of chemotherapeutic drugs against bladder cancer. But previous research has highlighted several different possible mechanisms for an anticancer action of lidocaine. These include reduced shedding from the cell surface of heparin-binding epidermal growth factor (EGF)-like growth factor (which is known to be involved in tumor progression and metastasis) as well as changes in intracellular calcium^12^, inhibiting the activity of the EGF receptor^15^, reduced calcium influx and downregulation of the expression of transient receptor potential cation channel subfamily V member 6 (TRPV6)^13^, inhibition of tumor necrosis factor-α-induced Src-activation and phosphorylation of intercellular adhesion molecule-1^14^, reduced activity of extracellular signal-regulated kinase (ERK) 1/2 and enhanced activity of p38 mitogen-activated protein kinase and c-jun N-terminal kinase (JNK)^16^. It may be that the mechanisms underlying the anticancer action of lidocaine vary between cancer cell types and that some of the above mechanisms are involved in the effects of lidocaine that we observed in BIU-87 cells. For example, reduced EGF receptor expression is associated with decreased growth, migration and invasion of BIU-87 cells^23^, while activation of ERK and JNK are associated with increased malignant behavior of BIU-87 cells^24–26^. This raises the possibility that inhibition of the activities of the EGF receptor, ERK and/or JNK may contribute to the effects of lidocaine observed in the present study. Another interesting possibility is that lidocaine might suppress bladder cancer cell proliferation through DNA demethylation^27^. A variety of tumor suppressor genes are methylated in bladder cancer^28^. Lidocaine can promote DNA demethylation and inhibit growth in breast cancer cells^29,30^, while procaine has been found to have similar effects in breast cancer cells^19^ and human hepatoma cells^31^. Two implicated tumor suppressor genes, retinoic acid receptor-β2 (RARβ2) and Ras association domain family-1A (RASSF1A)^29,30^, have also been implicated in the malignant behavior of bladder cancer cells^32–35^. Additional research will be required to establish whether any of the above pathways are involved in the inhibition of bladder cancer cell proliferation by lidocaine.

This study has some limitations. The cell based experiments used only one bladder cancer cell line (BIU-87), the action of lidocaine in other cell lines needs to be investigated in future to support these results. We also did not investigate the effects of combination therapy with pirarubicin or SFN in the animal study, as this would have required more animals. If the results of this study are considered important enough these experiments may be considered in future.

In conclusion, lidocaine was able to enhance the antiproliferative actions of MMC, pirarubicin and SFN on BIU-87 cells. In addition, in mice with orthotopic bladder cancer, the combination of MMC and lidocaine prolonged survival and reduced tumor size. Postoperative intravesical instillation of lidocaine in combination with other chemotherapeutic agents could potentially be more effective than monotherapy in the treatment of bladder cancer. Further studies are merited to explore this possibility.

## Materials and Methods

### Cell line

Human bladder cancer BIU-87 cells (a kind gift from Professor Xiaofeng Yang of the Department of Urology, our hospital, China) were cultured in RPMI-l640 medium (Gibco, Thermo Fisher Scientific, Waltham, MA, USA) supplemented with 0.05 g/L streptomycin (North China Pharmaceutical Group Corp., Shijiazhuang, China), 0.05 g/L penicillin (North China Pharmaceutical Group Corp., Shijiazhuang, China), 0.8 g/L NaHCO_3_ (Tianjin DaMao Chemical Reagent Company), 3.6 g/L HEPES (Sangon Biotech Co. Ltd, Shanghai, China) and 10% fetal bovine serum (Hangzhou Sijiqing Biological Engineering Materials Co. Ltd, Hangzhou, China). The cells were incubated at 37 °C in a humidified environment containing 5% CO_2_ (NU-5500E incubator, Nuaire, Plymouth, MN, USA). For the experiments, cells in the logarithmic growth phase were digested into suspension with 0.25% trypsin (Sangon Biotech Co. Ltd, Shanghai, China).

### Experimental animals

Forty female BALB/c nude mice (weighing 16–18 g; postnatal day 49–55) were purchased from Vital River Experimental Animal Technology Co. Ltd (Beijing, China; license number: SCXK (Beijing) 2012-0001). All animals were maintained in a specific pathogen-free environment with shavings as bedding (Xietong Medical Biological Engineering Co. Ltd, Jiangsu, China). The cages and bedding were sterilized at 121 °C for 20 min. All animals were allowed free access to food and water. The feed was purchased from Ke’aoxieli Feed Co. Ltd (Beijing, China). Drinking water was sterilized by ^60^Co irradiation.

The animal use certification number was SYXK (Jin) 2012-0001, and the study was approved by the Laboratory Animal Management Committee at the Shanxi Cancer Institute with reference to the National Research Council Guide for the Care and Use of Laboratory Animals in China. All applicable international, national, and institutional guidelines for the care and use of animals were followed.

### Measurement of BIU-87 cell proliferation *in vitro*

The methylthiazolyldiphenyl-tetrazolium bromide (MTT) assay was used to measure BIU-87 cell proliferation *in vitro*, and the influence of lidocaine on the antiproliferative effects of various agents was examined. The following experimental groups were used: control group (no drugs added); three separate lidocaine groups (application of either 1.25 mg/mL, 2.5 mg/mL or 5 mg/mL lidocaine; Beijing Yimin Pharmaceutical Co. Ltd, Beijing, China); MMC group (application of 0.66 mg/mL MMC; Zhejiang Haizheng Pharmaceutical Co. Ltd, Taizhou, China); pirarubicin group (application of 0.75 mg/mL pirarubicin; Shenzhen Wanle Pharmaceutical Co. Ltd, Wuhan, China); Su Fu’ning lotion (SFN) group (application of 0.0625 mg/mL SFN; prepared in the Pharmaceutical Department of our hospital, Taiyuan, China); and three separate combination groups (0.66 mg/mL MMC, 0.75 mg/mL pirarubicin or 0.0625 mg/mL SFN given in combination with either 1.25 mg/mL, 2.5 mg/mL or 5 mg/mL lidocaine). SFN, a lotion produced from sappan wood extract that has a brazilin content of above 52%, has been previously shown to inhibit bladder cancer cell growth both *in vitro* and *in vivo*^20^.

For the cell proliferation assays, BIU-87 cells (5 × 10^3^ cells/well) were seeded in 96-well plates (Bio Basic Inc., Markham, ON, Canada) containing 100 µL culture medium per well. The cells were incubated at 37 °C in a humidified environment containing 5% CO_2_. The culture medium was removed 24 h after cell adhesion and 200 µL of fresh medium containing the appropriate drug or combination of drugs (i.e. lidocaine, MMC, pirarubicin or/and SFN) was added; for the control group, 200 µL of fresh medium only was added.

MTT solution (5 mg/mL, Solarbio Life Sciences, Beijing, China) was added to each well 2 h after drug application, and the cells were incubated in MTT solution for 4 h. Then, the medium was removed and 150 µL DMSO (Tianjin Fuyu Fine Chemical Co. Ltd, Tianjin, China) was added to each well. The plate was oscillated at low speed for 10 min (on a shaker) to fully dissolve the crystals. The absorbance (optical density value, OD) at a wavelength of 570 nm was measured with a spectrophotometer (Tecan, Grödig, Austria). The cell inhibition rate for each group was calculated as: cell inhibition rate (%) = (1 − mean OD value in the experimental group)/mean OD value in the control group × 100%. Culture medium without cells was used to obtain a blank absorbance value. Five replicated wells were studied for each group.

### Establishment of an animal model of bladder cancer using orthotopic transplantation of bladder cancer cells

Animals were anesthetized by intraperitoneal injection of 60 mg/kg sodium pentobarbital and the hypogastrium was disinfected routinely. A 24-gauge venous indwelling needle cannula coated with liquid paraffin was slowly inserted into the bladder lumen through the urethral orifice to extract the urine. A core needle with deflection angle was slowly inserted into the sleeve with one hand fixing the sleeve and the other hand applying pressure to the core needle. The core needle was rotated for 8 rounds and then withdrawn. 100 μL of bladder tumor cell suspension containing 2 × 10^6^ BIU-87 cells was immediately injected into the bladder lumen. The sleeve was then pulled out slowly. The animals were positioned at 15–30° of hip elevation. Attention was paid to the animals’ body temperature until the animals had recovered naturally.

### Effect of bladder irrigation with lidocaine or/and MMC on orthotopically transplanted bladder tumor

32 female BALB/c nude mice were randomly divided into four experimental groups (8 animals in each) to receive intravesical instillation of drug(s)/vehicle 24 h after inoculation. The four experimental groups were as follows: vehicle group (intravesical instillation of water); MMC group (intravesical instillation of 0.2 mg/mL MMC); MMC/high lidocaine group (intravesical instillation of 0.2 mg/mL MMC plus 5 mg/mL lidocaine); and MMC/low lidocaine group (intravesical instillation of 0.2 mg/mL MMC plus 2.5 mg/mL lidocaine). For intravesical instillation, the animals were anesthetized by intraperitoneal injection of 60 mg/kg sodium pentobarbital, and the urethral orifice was partially disinfected. 100 µL of the appropriate solution was infused into the bladder lumen of each mouse, once weekly for a total of 3 instillations. Attention was paid to the animal’s body temperature from the time of drug infusion to the time at which the animal woke up. The doses of MMC and lidocaine used in the animals were calculated according to the dosages used in humans.

After intravesical administration of agents, the animals were maintained under normal conditions and their activities were observed. Animal weight, feed intake, water intake and survival were monitored regularly and recorded. The development of hematuria and the formation of bladder tumor were studied. The total observation time was 60 days, a survival time >60 days was recorded as 60 days. Anatomic dissection of the animal was performed immediately after its death. Bladder tumor formation and metastasis were observed, and the bladder was extracted and weighed (wet weight). To quantify the effects of MMC, lidocaine or their combination, tumor inhibition rate and survival extension rate were calculated for each experimental group, relative to the vehicle group, as follows:

$$\begin{array}{rcl}{\rm{Tumor}}\,{\rm{inhibition}}\,{\rm{rate}} & = & (\mathrm{average}\,{\rm{tumor}}\,{\rm{weight}}\,{\rm{in}}\,{\rm{the}}\,{\rm{vehicle}}\,{\rm{group}}\\  &  & -\,\,{\rm{average}}\,{\rm{tumor}}\,{\rm{weight}}\,{\rm{in}}\,{\rm{the}}\,{\rm{experimental}}\\  &  & \times \,\mathrm{group})/\mathrm{average}\,{\rm{tumorweight}}\,{\rm{in}}\,{\rm{the}}\,{\rm{vehicle}}\,{\rm{group}}\times \mathrm{100} \% ;\end{array}$$

$$\begin{array}{rcl}{\rm{Survival}}\,{\rm{extension}}\,{\rm{rate}} & = & (\mathrm{average}\,{\rm{survival}}\,[{\rm{days}}]\,{\rm{in}}\,{\rm{the}}\,{\rm{experimental}}\,{\rm{group}}\\  &  & -\,{\rm{average}}\,{\rm{survival}}\,[{\rm{days}}]\,{\rm{in}}\,{\rm{the}}\,{\rm{vehicle}}\mathrm{group})\\  &  & /\mathrm{average}\,{\rm{survival}}[{\rm{days}}]\,{\rm{in}}\,{\rm{the}}\,{\rm{vehicle}}\,{\rm{group}}\times \mathrm{100} \% {\rm{.}}\end{array}$$

### Statistical analysis

SPSS17.0 statistical software (SPSS Inc., Chicago, IL, USA) was used for the statistical analysis. Cell inhibition rate and tumor suppression rate were described as percentages (%). Survival time and bladder wet weight are presented as the mean ± standard deviation. Single factor analysis of variance was used for multi-group comparisons. The least significant difference (LSD) t*-*test was used when the variance was equal, while Dunnett’s T3 test was used when the variance was unequal. *P* < 0.05 was taken to indicate a statistically significant difference.

### Data availability

The data set supporting the results of this article are included within the article.
